# Case of Supposed Pigmentary Deposit in the Cortical Substance of the Entire Encephalon

**Published:** 1876-09

**Authors:** A. W. Hagenbuch

**Affiliations:** Assistant Physician Cook Co. Insane Asylum


### A CASE OF SUPPOSED PIGMENTARY DEPOSIT IN THE CORTICAL SUBSTANCE OF THE ENTIRE ENCEPHALON.

By A. W. HAGENBUCH, M. D.,

Assistant Physician Cook Co. Insane Asylum.

Magdelene Fischer, German, aged twenty-four years, single, was admitted to the asylum June 3d, 1875, with acute mania, which she had had for seven weeks, although she was thought by her friends to have acted peculiarly for several months prior to this period. Upon admission she was well nourished; heart and lung sounds normal; she was violent and very refractory, refusing, although perfectly able, to walk to her ward. After a few days she became somewhat melancholic, but occasionally was very violent, once even attacking one of the attendants, fighting desperately. She gradually became very quiet, and would sit for hours in one position and speak very little. Occasionally she sang snatches from the operas. She soon subsided into a profound stupor, sitting for hours in one position and never speaking. After a few weeks she gradually grew worse, and about March 1st, 1876, she began voiding her feces and urine in bed. There was not a continuous dribbling of urine or discharge of feces, but perfect evacuations several times in the day. She would sit for a half day with her head resting on her knees, and had to be fed and cared for like a child. She took to bed about April 13th; her physiognomy was remarkable, her face was a perfect blank, her eyes had an expressionless look about them, such as is rarely seen; she reminded me very strongly of a pigeon after both cerebral hemispheres are removed. A loud noise in her immediate neighborhood would startle her; she would make one or more sudden, spasmodic movements, then gradually subside to her former condition. She became very much emaciated. She seemed to be totally unconscious of pain, even a strong electric current would be born without the slightest manifestation of pain, although reflex movements seemed to be perfect, as was proven by irritating the soles of her feet; they would be immediately and spasmodically drawn upwards and removed from the cause of irritation. The case was regarded by me as a typical one of acute mania, terminating in complete dementia. The last few days of her life she had dysphagia; there seemed to be some paralysis of the pharyngeal muscles. During the acute attacks the temperature was 102° to 105° F.; during the intervals, 99° to 101° F.

She died May 2d, 1876. A *post mortem* examination was made 14 hours after death. The scalp was found very thin, and so distensible that the anterior flap might have been removed and the posterior one could have been easily stretched so as to take its place. The skull proper was thick — no irregularities upon its internal surface; dura mater very thick and pale; arachnoid about normal; pia mater very much thickened and congested, some of the bloodvessels having a tortuous course and resembling varicose veins. Weight of entire encephalon (including membranes) 43½ ounces. The external surface of the entire brain was very much darker than normally; it was almost black.

Upon removing a portion of the upper surface of the brain corresponding to the *centrum ovale minus*, the knife met with some resistance, producing a grating sensation to the hand, and with an audible sound. Particles of sandy substance had been deposited in the substance of brain. The cortical layers were almost black. Unfortunately the specimen was not examined microscopically. I am inclined to think the deposit was pigment. This deposit was found in the gray substance of the entire encephalon and spinal cord as low down as examined, about the fifth cervical vertebra, and sclerotic coats of both eyes. All the other internal organs were apparently healthy, with the exception of a small tubercular deposit in the apex of right lung.

